# Phylogenetic signature and prevalence of natural resistance-associated substitutions for hepatitis C virus genotypes 3a and 3b in southwestern China

**DOI:** 10.1016/j.jve.2022.100071

**Published:** 2022-06-15

**Authors:** Xiaoqing Liu, Zhiwei Chen, Qiao Tang, Peng Hu

**Affiliations:** Department of Infectious Diseases, Institute for Viral Hepatitis, The Key Laboratory of Molecular Biology for Infectious Diseases, The Second Affiliated Hospital of Chongqing Medical University, Chongqing, China

**Keywords:** Resistance-associated substitutions, Hepatitis C virus, Drug resistance, Phylogenicity, DAA, direct-acting antiviral, HCV, hepatitis C virus, RAS, resistance-associated substitution, SVR, sustained viral response

## Abstract

**Background:**

Patients infected with hepatitis C (HCV) genotype (GT) 3, especially GT3b, are still difficult to cure. GT3b is more common than GT3a in southwestern China. Here we aimed to investigate the prevalence of naturally occurring RASs in HCV GT3 in southwestern China and performed phylogenetic analysis.

**Methods:**

Serum samples were collected from patients with HCV GT3 infection. Sanger sequencing was used to validate resistance-associated substitutions (RASs). Phylogenetic analysis was performed using MEGA X and the observed-minus-expected-squared algorithm was used to analyze amino acid covariance.

**Results:**

A total of 136 patients were enrolled, including 41 HCV GT3a and 95 GT3b infected patients. In the NS5A region, the proportion of RASs found in GT3b (99%) was notably higher than in GT3a (9%). In the NS3 region, RASs prevalence in GT3b (5%) was lower than in GT3a (24%). NS5B-specific RASs were rare. Both the NS5A30k and L31 M substitutions occurred in 96% of GT3b sequences. The A30K + L31M combination was found in 94% of GT3b isolates, however, there were no A30K or L31M mutations observed in the GT3a sequence.

**Conclusions:**

Significant differences were observed between HCV GT3a and GT3b in terms of RAS prevalence. The origin of GT3a appears to be more diverse compared with GT3b in southern China. Studies specifically aimed at HCV GT3b infection should be initiated to gain more insight into this subtype.

## 1 Introduction

Hepatitis C virus (HCV) has infected approximately 71 million people worldwide, with approximately 1.75 million new infections each year according to the World Health Organization.^1^ Owing to the low fidelity of the NS5B polymerase and high replication rate, HCV has a substantial degree of genetic diversity. Eight genotypes and 105 subtypes have been found thus far.^2^, ^3^, ^4^, ^5^ HCV displays around 15% and 30% divergence at the subtype and genotype levels, respectively.^3^ HCV infection is one of the important causes of cirrhosis and hepatocellular carcinoma (HCC). Compared with other genotypes, patients with HCV genotype 3 (GT3) have a greater risk of significant fibrosis, cirrhosis and HCC than those with other genotypes,^6^, ^7^, ^8^ especially subtype 3b.^9^

The number of patients infected with HCV genotype 3 (GT3) is second only to genotype 1 (GT1), accounting for approximately 30.1% of cases worldwide.^10^ Owing to population mobility and changes in transmission pathways, the distribution pattern of HCV genotypes in China has evolved. Except for the northwest and central regions, a significant decrease in the proportion of GT1b has been observed. Moreover, the occurrence of GT3 and GT6 infections has risen significantly in the southern and southwestern parts of China.^11^, ^12^, ^13^ From the southwest to the northeast of China, the proportion of HCV GT3 infections has gradually decreased, and GT3b infections are now slightly higher than GT3a ones in the southwest.^14^ However, in North America, Europe, and Oceania, the proportion of GT3b infections is less than 1% of GT3.^15^ Therefore, as most studies have been conducted on GT3a infection, more attention needs to be paid to the GT3b subtype.

Patients infected with HCV GT3 have become the most difficult ones to treat in the direct antiviral agent (DAA) era,^16^ even with pangenotypic DAAs. With the recent advent of pangenotypic DAAs, the HCV sustained virologic response (SVR) rates can reach more than 95%, regardless of the presence of cirrhosis or RASs.^17^^,^^18^ However, compared with other genotypes, GT3 has a lower SVR, and the presence of baseline RASs may impair the DAA treatment response, especially in patients with cirrhosis.^19^, ^20^, ^21^, ^22^ A phase III clinical study, mainly focused on the Chinese population, reported that patients with GT3b, especially those with cirrhosis, had a decreased SVR rate (76%, 32/42).^20^

The RAS prevalence varies between subtype^15^ and region.^23^ Understanding the proportion of natural RASs could optimize the selection of the treatment regimens and durations, especially in the absence of sequencing for each patient. However, to the best of our knowledge, the distribution pattern of naturally occurring RASs in HCV GT3 in southwest China is still unknown. The main purpose of this study was to assess the prevalence of naturally occurring RASs in DAA-naive patients with HCV GT3 infection.

## 2 Materials and methods

### 2.1 Patients and samples

From January till July 2019, we collected 136 serum samples from DAA treatment-naive HCV GT3 patients from the Second Affiliated Hospital of Chongqing Medical University. The HCV subtype was confirmed by RT-PCR and sequencing of the HCV phylogenetic analysis. The study was approved by the Ethics Committee of the Second Affiliated Hospital of Chongqing Medical University, and written informed consent was obtained from each participant.

### 2.2 RNA extraction, amplification and sequencing of the HCV NS3, NS5A and NS5B regions

The detailed procedures of RNA extraction, amplification and sequencing were performed as previously described.^24^ Briefly, total viral RNA was extracted from 140 μL serum samples using the MiniBest Viral RNA/DNA Extraction Kit (TaKaRa Bio, Kusatsu, Japan); reverse transcription was performed using a PrimeScript RT Reagent Kit with Genomic DNA Eraser (TaKaRa) following the manufacturer's instructions; portions of the HCV NS3, NS5A and NS5B genes were amplified using PrimeSTAR HS DNA Polymerase (TaKaRa) in a nested PCR. The primers used in this study are shown in Table S1. The extension time in the PCR cycle was modified by the length of the target gene fragment. The DNA product was sequenced by direct Sanger sequencing using an automatic sequencer (ABI Prism 3100 genetic analyser; Thermo Fisher Scientific, Waltham, MA, USA).

### 2.3 RAS analysis

Sequence fragments were assembled using BioEdit 7.2.5 software, aligned with the reference sequence (GU814263) using the Clustal W algorithm and subsequently checked manually. All sequences were trimmed to include the largest common region encompassing amino acid positions NS31-233, NS5A1-233 and NS5B157-330. RASs in this study only include pangenotypic drug-related variants as recommended for the treatment of HCV patients. Both RASs observed during in vitro studies and those observed at virological failure in HCV GT3 patients were reported.

### 2.4 Phylogenetic analysis

Sequences of the NS5B region obtained from the present study and reference sequences retrieved from the GEO database were used to conduct phylogenetic analysis. The model test function of MEGA X was used to select the best-fitting substitution model with the one with the smallest AICc considered to be optimal. The Maximum likelihood estimation was used to infer evolutionary history. By applying the Neighbor Joining and BIONJ algorithms to the pairwise distance matrix estimated using the Maximum Compound Likelihood (MCL) method, the initial tree for heuristic search was automatically obtained. The tree was drawn to scale, and the branch lengths measured by the number of substitutions at each site. The codon positions included were 1st+2nd+3rd + Noncoding. Phylogenetic trees were constructed by MEGA X^25^ and visualized by FigTree version 1.4.4. A bootstrap analysis with 500 replicates was used to assess the reliability of the cluster descending from each node.

### 2.5 Amino-acid covariance analysis

Sequences of all NS3, NS5A and NS5B genes that were successfully detected in this study were aligned and translated into amino acid sequences using BioEdit 7.2.5 software. We used then the observed-minus-expected-squared (OMES) algorithm to identify covarying pairs. An OMES score of 0.5 was considered as the cutoff as it could retain the maximum amount of potentially informative data without the influence of noise, as described by others. Cytoscape 3.7.2 was used to visualize amino acid covariance networks.

### 2.6 Statistical analysis

Data were analyzed with the R3.5.2 software. Continuous variables are expressed as medians and interquartile range (IQRs), whereas categorical variables are presented as numbers (percentages). Categorical variables between HCV GT3a and GT3b were compared using the chi-square test. Continuous variables between groups were compared using the Mann–Whitney test. A p value of <0.05 was considered to be statistically significant.

## 3 Results

### 3.1 Baseline characteristics of the study population

We recruited a total of 136 patients, among whom 41 had GT3a and 95 GT3b infections. The NS3, NS5A, and NS5B genes were successfully amplified and sequenced from 38, 33 and 29 GT3a patients and 80, 81 and 64 GT3b patients, respectively. Their baseline characteristics are shown in Table 1. More GT3a than GT3b infections were present in younger individuals. Except for age, there was no significant difference between the two groups.

**Table 1:** Baseline characteristics of the study population.

	3a	3b	p-value
Number	41 (30%)	95 (70%)	
Age (years)	43 (35–48)	48 (42–54)	**0.005**
Male/female	25/16	61/34	0.868
Alb(g/L)	42.1 (39.5–45.0)	42.2 (37.2–44.4)	0.522
ALT (IU/L)	84 (54–123)	81 (42–131)	0.706
AST (IU/L)	60 (45–97)	73 (46–106)	0.678
GGT (IU/L)	43 (34–162)	62 (47–119)	0.220
TBIL (μmol/L)	13.3 (9.6–23.3)	11.7 (8.6–17.6)	0.170
DBIL (μmol/L)	5.5 (3.6–9.8)	5.0 (3.6–7.1)	0.313
PLT count ( × 10^9/L)	157 (108–220)	148 (87–197)	0.397
APRI	1.0 (0.5–2.5)	1.3 (0.7–2.7)	0.443
HCV RNA (log 10 IU/mL)	6.4 (5.8–6.8)	6.2 (5.5–6.7)	0.270

Data are expressed as median (IQR) or n (%). Significant bold are shown in bold.

Abbreviations: ALT, alanine aminotransferase; AST, aspartate aminotransferase.

Alb, albumin; PLT, platelet; GGT, gamma-glutamyl transferase; TBIL, total bilirubin; DBIL, direct bilirubin; APRI, Aspartate aminotransferase-to-Platelet Ratio Index.

### 3.2 Significant differences between GT3a and GT3b infections in the prevalence of RASs

The criteria for the selection of GT3-specific RASs were obtained from the literature.^17^^,^^26^, ^27^, ^28^ A comprehensive overview of RASs in protease inhibitors, NS5A inhibitors, and nucleoside NS5B inhibitors appears in Table S2. The prevalence of amino acid polymorphisms in resistance-associated positions can be found in Table S3.

Overall, NS3 RASs were detected in 9 of 38 (24%) GT3a and 4 of 80 (5%) GT3b sequences as shown in Fig. 1. Among patients with HCV GT3a infection, 9 of 38 (24%) isolates had a substitution at position 166. No resistant mutation occurred at the same position in GT3b sequences. Variants at position 168 could confer high-level resistance to currently approved NS3 inhibitors for the treatment of GT3, and 1 of 80 (1%) GT3b patients had a Q168L substitution.

**Fig. 1:**
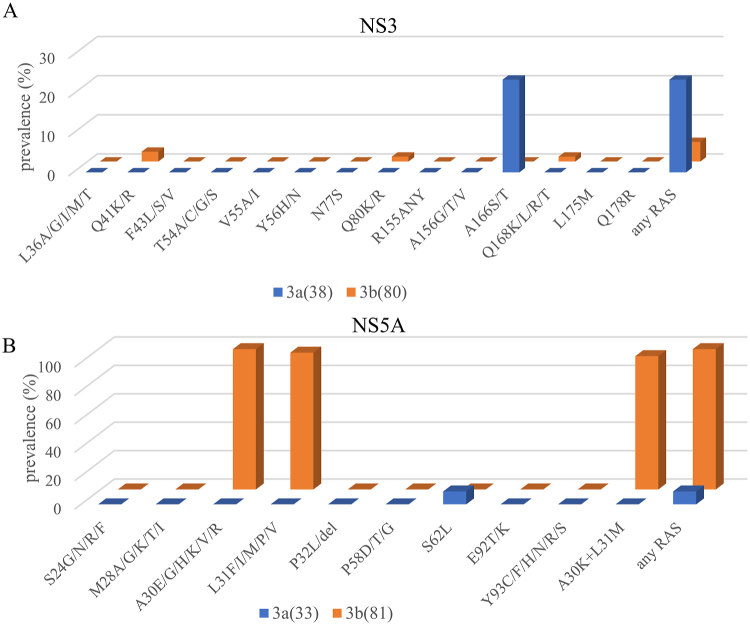
**Prevalence of RASs in HCV GT3.** Frequency of RASs in the NS3(A) and NS5A regions(B) in hepatitis C GT3a and GT3b subtypes. The criteria for the selection of GT3-specific RASs were obtained from the literature.^17^^,^^26^, ^27^, ^28^

A significant difference was observed in the distribution pattern of NS5A RASs between GT3a and GT3b patients, which is shown in Fig. 1. In this study, no NS5A RAS was observed in GT3a isolates except for S62. But in patients infected with GT3b, 96% (78/81) of sequences harbored NS5A A30K and L31M mutations, 2% (2/81) of patients harbored the A30R substitution, and the A30K + L31M combination was detected in 94% of individuals. No RASs were observed in other positions.

As expected, the prevalence of NI NS5B RASs was rare. Currently, the S282T substitution is the only variant known to reduce susceptibility to sofosbuvir (SOF) in vitro,^29^ however, we did not detect it in this study. The only RAS detected was the L159F substitution in GT3b isolates. There was no RAS detected in GT3a isolates. L159F, however, is not associated with reduced SOF susceptibility in vitro*.*^30^ The L159F substitution is associated with lower SVR rates in shorter duration (<24 weeks) regimens containing SOF and ribavirin (RBV), but not in a combined regimen targeting both NS5A and NS5B or with pegylated interferon.^31^

Among the 78 patients who were tested for natural resistance of all 3 genes, 26 tested positive for GT3a and 52 for GT3b. No GT3a isolates contained RASs in multiple regions. For GT3b, although the prevalence of RASs in the NS3 and NS5B regions was low, due to the high prevalence of NS5A RASs, 4% of the isolates had multiple RASs in the NS3+NS5A region, and 2% had multiple RASs in the NS5B + NS5A region.

### 3.3 The origin of HCV GT3a appears to be more diverse compared with GT3b

To investigate the genetic relationship between HCV GT3a and GT3b circulating in southwestern China, phylogenetic trees were constructed with the NS5B sequences amplified in our study and two reference sequences (3a-D17763, and 3b-D49374). Sequences within the same subtype clustered closely together (Fig. S1), suggesting a great diversity between GT3a and GT3b isolates. We can clearly observe that most GT3a sequences were divided into two clusters, while GT3b sequences were relatively concentrated.

**Fig. 2:**
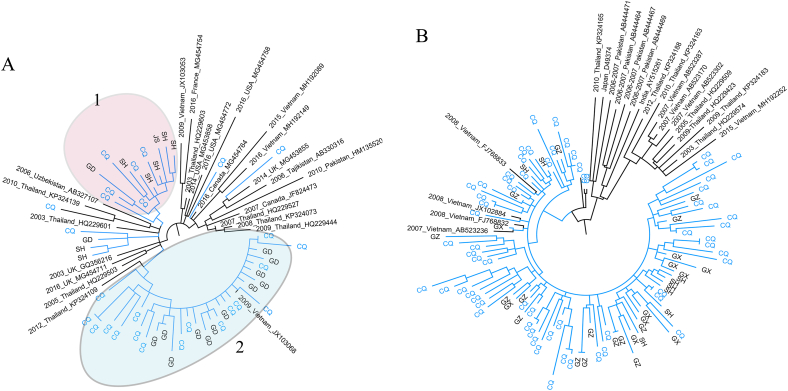
Phylogenetic tree of the NS5B region. Circular maximum likelihood trees constructed by GT3a NS5B (A)and GT3b NS5B partial sequences (B). Isolates originating from abroad are indicated by collection date, followed by accession number. Lines marked in blue represent domestic isolates, which are indicated by geographic codes: CQ=Chongqing, GD = Guangdong, GX = Guangxi, SH=Shanghai, and JS = Jiangsu. The bar at the bottom of the Figure shows the scale for nucleotide substitutions per site. (For interpretation of the references to colour in this figure legend, the reader is referred to the Web version of this article.)

We further analyzed the phylogenetic relationship between local GT3 sequences and reference sequences obtained from GenBank®. After sequence alignment and trimming, the 323-bp and 324-bp fragments of the NS5B region for GT3a and GT3b were used for analysis, respectively. A total of 71 GT3a isolates detected in our study and retrieved from GenBank® were used to construct the ML tree. Most Chinese sequences were clustered into two groups, as shown in Fig. 2A. Cluster 1 contained sequences from southwestern and southern China and was closely related to isolates from Vietnam and Thailand, suggesting a close genetic relationship between patients from southwestern and southern China and patients from Vietnam and Thailand, while cluster 2 sequences were mainly from the southwestern and eastern coastal areas of China. The distinction between these two clusters may indicate that HCV in Chongqing comes from two different areas. Fig. 2B displays a GT3b ML tree consisting of 64 sequences from our study and 54 reference sequences from around the world. Sequences from China formed one cluster and were closely related to some isolates from Vietnam, but they were clearly separated from those of other Asian countries (Japan, India, Pakistan, and Thailand). Sequences from Guangxi, Guangdong and Shanghai which are located in the southern, southern coastal, and eastern regions of China, respectively, were scattered throughout this cluster.

### 3.4 Amino acid covariance networks of the NS3, NS5A and NS5B genes

**Fig. 3:**
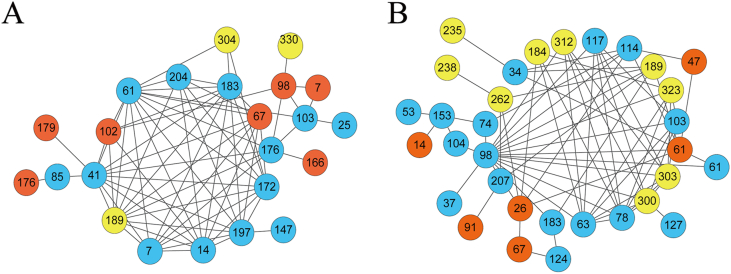
**Amino acid covariance networks for NS3, NS5A and NS5B sequences in HCV GT3.** Amino acid covariance networks were constructed by partial GT3a sequences (A) and partial GT3b sequences (B), respectively. Each node represents one amino acid position. The position of the numbered amino acid residue is provided relative to the first amino acid of the NS3, NS5A, or NS5B region. Orange nodes are within the NS3 region, blue nodes are within the NS5A region, and yellow nodes are within the NS5B region. (For interpretation of the references to colour in this figure legend, the reader is referred to the Web version of this article.)

To further understand the covariance relationship and detect all possible amino acid covariance pairs among the NS3, NS5A and NS5B region, a covariance network was constructed. Each node contains a covarying amino acid, and each line shows the covariance between two positions. Significant differences between GT3a and GT3b exist. As shown in Fig. 3, there were 23 covarying positions and 138 edges in GT3a, and 32 covarying positions and 142 edges in GT3b. The amino acid positions that formed the nodes in the networks were very different between GT3a and GT3b, with only five shared nodes. The most highly connected node lied in position 166 in the NS3 region in GT3a, but in position 98 in the NS5A region. Only one resistance-associated site in GT3a was involved in the network, while there were no such sites in GT3b.

## 4 Discussion

Even when treated with pangenotypic regimens, patients with GT3 and cirrhosis are still difficult to cure.^32^ To reach the goal of eliminating viral hepatitis as a major public health threat by 2030 as proposed by the World Health Organization, it is important to further investigate the treatment of HCV GT3. Sofosbuvir/velpatasvir (SOF/VEL), sofosbuvir/velpatasvir/voxilaprevir (SOF/VEL/VOX), glecaprevir/pibrentasvir (GLE/PIB), sofosbuvir/daclatasvir (SOF/DAC) and SOF/ribavirin have been approved for the treatment of HCV GT3 infections in mainland China. Recently, with the sharp reduction in the price of SOF/VEL, more people can afford these treatment costs. It is becoming increasingly urgent to understand the epidemic situation of RASs in order to optimize treatment regimen and course.

A total of 136 patient samples were collected in our study, and the percentages of patients with GT3a and GT3b infection were 30% and 70%, respectively. Compared with a study conducted by Y Chen et al.^14^ 3 years ago, the proportion of GT3b isolates in Chongqing has increased.

In the NS3 region, the prevalence of GT3a RASs is clearly higher than in GT3b RASs. Two of the most common GT3a RASs were A166S and A166T for which the prevalence rate was 24% in total. The A166T confers low level GLE resistance,^28^ and the A166S is the most common mutation among patients who fail the GLE/PIB regimen.^33^ Patients with an A166S mutation showed a lower SVR rate (82%, 14/17) in a pooled analysis.^17^ In the ENDURANCE-3 trial of patients treated with GLE/PIB for 8 weeks, GT3 infected non-cirrhotic patients with NS3 polymorphisms had lower SVR12 rates than patients without these polymorphisms (86% versus 98%).^22^

Natural NS5A RASs were associated with a decreased SVR with a 12-week regimen of SOF/DAC or SOF/VEL in GT3 infected patients with cirrhosis.^21^^,^^34^, ^35^, ^36^ In the NS5A region, for patients infected with GT3b, 99% (80/81) of isolates were found to have mutations at position 30 or 31, and 94% (76/81) of the GT3b isolates harbored the A30K + L31M combination. Nevertheless, no RAS was observed across GT3a isolates at positions 30 and 31. This result is similar to a previous study by Jie Lu et al.^37^ based on the general Chinese population. In an in vitro study, the combination of A30K + L31M showed a >10,000-fold increase in the EC50 to VEL and DAC. However, resistance to PIB was observed at lower levels (>20-fold increase in EC50).^38^ Moreover, a difference in efficacy of available pangenotypic regimens between HCV GT3a and GT3b was also observed in clinical trials. In a recent study evaluating the efficacy of SOF/VEL for patients with HCV infection conducted in Asia, SVR12 was achieved in 95% of patients with GT3a and 76% (32/42) in those with GT3b. Of all the patients with GT3b, 89% (25/28) of those without cirrhosis and 50% (7/14) of those with cirrhosis achieved SVR12 with 12 weeks of SOF/VEL.^20^ To our knowledge, there are no clinical studies about the therapeutic effect of G/P or SOF/VEL/VOX in HCV GT3b infection. In patients with GT3 receiving G/P, the presence of the A30K decreased the SVR12 rates (36/44) significantly.^39^ Currently, guideline-recommended treatments do not distinguish between GT3a and GT3b. However, this information may be important for choosing treatment regimens. Our study showed that patients with GT3b seem to be more difficult to treat than those with GT3a, which is consistent with other research findings.^37^^,^^38^ Given that adding ribavirin to SOF/VEL for GT3 patients with cirrhosis could increase the SVR rate,^34^^,^^35^ this combination may be a reasonable choice for patients with GT3b. However, the exact efficacy of the protocol approved for the treatment of GT3 HCV with GT3b needs further study.

We note that no Y93H mutation was detected in either GT3a or GT3b infections. The Y93H was not detected in GT3b, despite being reported by several studies,^15^^,^^37^^,^^38^ which may indicate that we do not need to consider Y93H when selecting a treatment regimen due to the low prevalence of GT3b. However, compared with other studies, the lower prevalence of Y93H in GT3a in our study needs to be confirmed with a larger sample size.

SOF is the only NS5B nucleoside inhibitor (NI) that is approved for the treatment of GT3 HCV. The L159F substitution was enriched in GT3 patients who had failed SOF-containing regimens^40^ and was the only NS5B NI mutation detected in this study. Our study confirmed that resistance-associated substitutions in the NS5B region are rare.

Our phylogenetic analysis revealed that the origin of GT3a appears to be more diverse than that of GT3b, and the global geographic distribution of GT3a and GT3b may explain this difference. The great diversity between GT3a and GT3b shown in the GT3 NS5B ML tree and covariance relationships, confirm our previous conclusion that the treatment of patients with these two subtypes should be different.

Although our study showed significant differences between HCV GT3a and GT3b, our study has several limitations. First, due to the short lengths of our sequenced samples, it is impossible to conduct further in-depth evolutionary analysis and amino acid covariance analysis. Second, we do not have clinical treatment outcomes from our enrolled patients. However, several studies have shown that patients with GT3b have a lower SVR.^20^ Finally, this is a single-center study, and whether our findings found can be applied to other regions requires further investigation.

In conclusion, the treatment of patients infected with HCV GT3b and cirrhosis seems to be a greater challenge. Owing to the limited data on GT3b and its high prevalence in Asia, further insight into the treatment of GT3b should it may be beneficial in terms of treatment outcome.

## Funding

This work was supported in part by the National Science and Technology Major Project of China [2017ZX10202203-008].

## Authors' contributions

Conceptualization, P·H.; Methodology, X.Q.L., Z.W·C., Q.T.; Software, X.Q.L., Z.W·C.; Validation, X.Q.L., Z.W·C.; Formal Analysis, X.Q.L., Z.W·C.; Investigation, X.Q.L., Z.W·C.; Resources, P·H., X.Q.L., Z.W·C., Q.T.; Data Curation, X.Q.L., Z.W·C.; Writing – Original Draft Preparation, X.Q.L; Writing – Review & Editing, X.Q.L., P·H.; Visualization, X.Q.L., Z.W·C; Supervision, P·H.; Project Administration, P·H.; Funding Acquisition, P.H. All authors contributed to the article and approved the submitted version.

## Institutional review board statement

The study was conducted according to the guidelines of the Declaration of Helsinki, and approved by the ethics committee of the Second Affiliated Hospital of Chongqing Medical University (2019 (30)).

## Informed consent statement

Not applicable.

## Data availability statement

The datasets generated during the current study are available from the corresponding author on reasonable request.

## Declaration of competing interest

The authors declare that they have no known competing financial interests or personal relationships that could have appeared to influence the work reported in this paper.

## Figures and Tables

**figs1:**
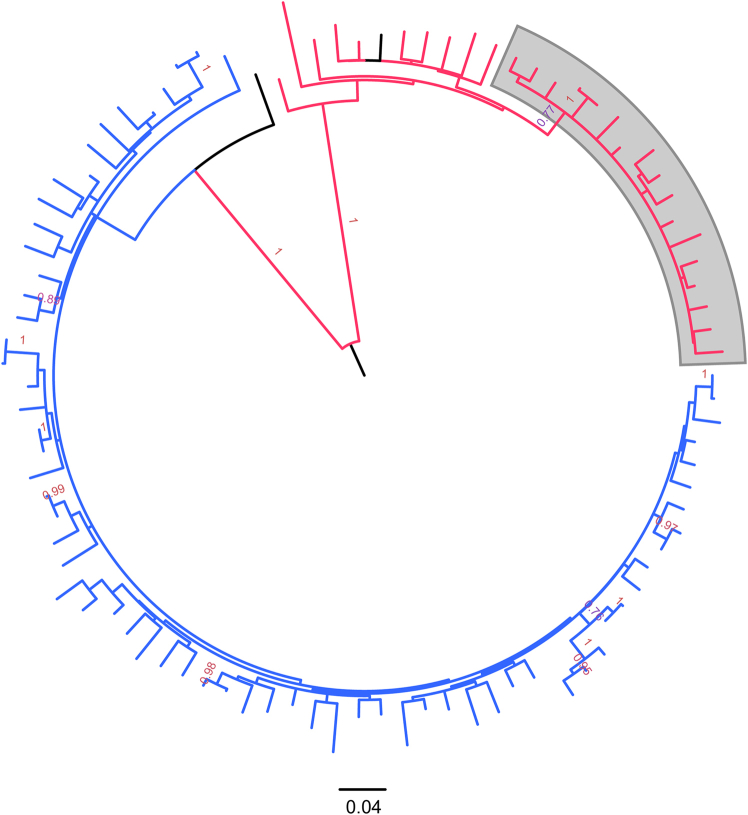


## Abbreviations

ALT: alanine aminotransferase
AST: aspartate aminotransferase
Alb: albumin
DAA: direct-acting antiviral
DBIL: direct bilirubin
TBIL: total bilirubin
GGT: gamma-glutamyl transferase
HCV: hepatitis C virus
NS: nonstructural protein
NI: nucleoside inhibitor
PLT: platelet;
RAS: resistance-associated substitution
SVR: sustained viral response
APRI: Aspartate aminotransferase-to-Platelet Ratio Index
