# Supplementary material for: Phylogenetic signature and prevalence of natural resistance-associated substitutions for hepatitis C virus genotypes 3a and 3b in southwestern China
Source: J Virus Erad. 2022 Jun 15;8(2):100071. doi: 10.1016/j.jve.2022.100071 (PMC9218835; doi:10.1016/j.jve.2022.100071)
Supplement: Multimedia component 1 [file mmc1.doc]

**Supplementary Materials**

**Supplementary Tables**

**Table S1.** Amplification and sequencing primers for HCV NS3, NS5A, and NS5B genes in genotype 3a and 3b patients.

| Target gene | primers | 5'-3' sequences | |
| --- | --- | --- | --- |
|  |  | GT3a | GT3b |
| NS3 | NS3F out | GATACAGCRGCTTGYGGAGA | AGCCCGTCATCTTCAGTCCTAT |
|  | NS3R out | TATTGCGGAYGTTRGGGTCG | GCCAGCACTGTTAGCCTCAC |
|  | NS3F in | TATACGRTTRGGCCRTGAGGTG | TATCATCTGGGGTGCGGACA |
|  | NS3R in | ATGTTRGGGTCGATCCCRTA | CCAGTATGGAGGTGGCATCTTG |
| NS5A | NS5AF out | GCGGTACARTGGATGAACAG | GGAGCAGTACAGTGGATGAACAG |
|  | NS5AR out | AGCGGACCAAGARTACGACA | GGAGCAAAGGAGGGTTGTAGTC |
|  | NS5AF in | GTGCARTGGATGAACAGRCT | GGGACTACAGTGGATGAACAGAC |
|  | NS5AR in | CCARGARTACGACATAGAGC | GCCTATGCGTTTGACAAGTGG |
| NS5B | NS5BF out | GTSTGGARGAGYTCTGGAAGAC | CCTACCTATCAGTCCACTCAGCA |
|  | NS5BR out | RGGCRGARTACCTRGTCATAGCCT | CCACCCGCTGTGATAAATGTCGT |
|  | NS5BF in | ACYATCATGGCTAARARYGAGGT | AAGGAGGTGAAGGAGCGAGC |
|  | NS5BR in | ACCTRGTCATAGCCTCCGTGAA | GGTCTGGTTTTGGTACGCACT |

**Table S2**. Summary of RASs in NS3, NS5A, NS5B regions

|  | **RASs** |
| --- | --- |
| **NS3** | L36A/G/I/M/T, Q41K/R, F43L/S/V, T54A/C/G/S, V55A/I, Y56H/N, N77S, Q80K/R, R155ANY, A156G/T/V, A166S/T, Q168K/L/R/T, L175M, Q178R |
| **NS5A** | S24G/N/R/F, M28A/G/T/I/K, A30E/G/H/K/V/R, L31F/I/M/P/V, P32L/del, P58D/T /G, S62L, E92T/K, Y93C/F/H/N/R/S |
| **NS5B** | L159F, E237G, S282T, V321A |

a summary of RASs to protease inhibitors (GLE, VOX), NS5A inhibitors (DAC,VEL,PIB) and nucleoside and NS5B inhibitors (SOF).

**Table S3**. Prevalence of baseline polymorphisms in NS3, NS5A, NS5B in HCV GT3 infected patients

| The frequency of NS5A substitution | | | | | |
| --- | --- | --- | --- | --- | --- |
| position | | 3a | | 3b | |
|  |  | substitution | Num (%) | substitution | Num (%) |
| NS5A | S24 | T | 1(3%) |  |  |
|  | M28 |  |  | L | 1(1%) |
|  | A30 | T | 1(3%) | **K** | 78(96%) |
|  |  | S | 1(3%) | **R** | 2(2%) |
|  | L31 |  |  | **M** | 78(96%) |
|  | P32 |  |  |  |  |
|  | P58 |  |  |  |  |
|  | S62 | **L** | 3(9%) | D | 5(6%) |
|  |  | P | 2(6%) | E | 72(89%) |
|  |  | T | 2(6%) | N | 2(2%) |
|  |  |  |  | P | 1(1%) |
|  |  |  |  | V | 1(1%) |
|  | E92 |  |  |  |  |
|  | Y93 |  |  |  |  |
| NS5B | L159 | P | 2(7%) | **F** | 1(2%) |
|  | E237 | A | 2(7%) |  |  |
|  | S282 |  |  |  |  |
|  | V321 |  |  | G | 1(2%) |
| NS3 | L36 |  |  |  |  |
|  | Q41 |  |  | **R** | 2(3%) |
|  | F43 |  |  |  |  |
|  | T54 |  |  |  |  |
|  | V55 |  |  |  |  |
|  | Y56 | F | 1(3%) |  |  |
|  | N77 |  |  |  |  |
|  | Q80 |  |  | **K** | 1(1%) |
|  | R155 |  |  |  |  |
|  | A156 |  |  |  |  |
|  | A166 | **T** | 3(8%) | G | 2(3%) |
|  |  | **S** | 6(16%) | P | 1(1%) |
|  | Q168 |  |  | **L** | 1(1%) |
|  | L175 |  |  |  |  |
|  | Q178 |  |  |  |  |

Bold words represent resistance-associated substitution.

**Supplementary Figures**

**Figure S1. Phylogeny of NS5B sequences.**

Circular maximum likelihood trees were constructed by GT3a and GT3b NS5B partial sequences determined in this study and 2 reference sequences(3a-D17763,3b-D49374) retrieved from GenBank. The lines marked in black represent reference sequences. The bar at the bottom of the figure shows the scale for nucleotide substitution per site.
